# Engineering tolerance to CLCuD in transgenic *Gossypium hirsutum* cv. HS6 expressing *Cotton leaf curl Multan virus*-*C4* intron hairpin

**DOI:** 10.1038/s41598-021-93502-3

**Published:** 2021-07-08

**Authors:** Mirza S. Baig, Sadia Akhtar, Jawaid A. Khan

**Affiliations:** 1 Department of Biosciences, Jamia Millia Islamia (Central University), Jamia Nagar, New Delhi, 110025 India; 2 Department of Molecular Medicine, Jamia Hamdard, Hamdard Nagar, New Delhi, 110062 India

**Keywords:** Microbiology, Plant sciences

## Abstract

Cotton leaf curl disease (CLCuD), caused by begomoviruses in combination with betasatellite molecule, has adversely affected cotton industry of Indian subcontinent. To devise a CLCuD-control strategy, RNAi-mediated approach was followed in this study. *Gossypium hirsutum* cv. HS6 plants were transformed with intron-hairpin RNAi (ihpRNAi-C4) construct carrying silencing suppressor *C4* gene of *Cotton leaf curl Multan virus* (CLCuMuV). Efficacy of the construct in imparting CLCuD resistance was evaluated in transgenic (T_0_, T_1_) cotton lines. Accumulation of CLCuMuV/betasatellite and attenuation of CLCuD symptoms in the transgenic lines were monitored at different times interval after virus inoculation. Northern hybridization revealed the expression of C4-gene derived siRNA. Expression of the ihpRNAi transcript was recorded higher in transgenic lines expressing siRNA which supposedly targeted the *C4* gene. A significant delay in detection of virus as well as betasatellite was observed in the transgenic lines. At 30 days post inoculation (dpi), none of the lines tested positive. At 45 dpi, however, it could be detected in few lines having much lower titre as compared to non-transformed control plants. Notably, till 60 dpi, no significant progression of the virus/betasatellite DNA was observed and the plants did not exhibit any characteristic CLCuD symptoms. A tolerance phenomenon leading to escape of CLCuD symptoms in the transformed cotton was described.

## Introduction

Cotton (*Gossypium hirsutum*) is the most important source of natural fibre for textile, paper and vegetable oil industries worldwide. India is the second largest producer as well as exporter of cotton^1^. Most commercially grown cotton (~ 90%) in India belongs to different cultivars of *G. hirsutum*, whereas other species like *G. arboreum*, *G. barbedense* and *G. harbaceum* collectively contribute about 10% of total cultivation^2^. Cotton leaf curl disese (CLCuD), caused by begomoviruses in association with betasatellite, is endemic in north-western states (Punjab, Haryana, and Rajasthan). It inflicts enormous losses to the crop by reducing yield and compromising fibre quality. All cultivated varieties of *G. hirsutum* in the Indian subcontinent are susceptible to distinct begomoviruses associated with CLCuD (BAC) and Cotton leaf curl Multan betasatellite (CLCuMB).

Presently, there are five BAC species belonging to single-stranded (ss), circular DNA viruses (family *Geminiviridae*). The most widespread are *Cotton leaf curl Multan virus* (CLCuMuV) and *Cotton leaf curl Kokhran virus* (CLCuKoV), while *Cotton leaf curl Bangalore virus*, *Cotton leaf curl Alabad virus*, *Cotton leaf curl Gezira virus* and *Papaya leaf curl virus* are less frequently encountered in the Indian subcontinent^3,4^. They are transmitted exclusively by whitefly (*Bemisia tabaci*) in a circulative, persistent manner. The monopartite CLCuMuV and CLCuKoV genomes in association with Cotton leaf curl Multan betasatellite molecule have been demonstrated to cause CLCuD^5,6^. The initial symptoms of CLCuD are characterized by darkening and thickening of leaf veins, subsequently causing upward (sometimes downward) curling of leaf, formation of cup-shaped leaf-like structures (enations) on the underside of the leaf. The infected plant becomes stunted due to reduced inter-nodal distance. Furthermore, flowering, formation and maturation of bolls are affected leading to lower yield and poor fibre quality^7^. Over the last three decades, CLCuD remains the most disastrous disease of cotton and a major limiting factor for its production in the Indian subcontinent.

**Figure 1:**
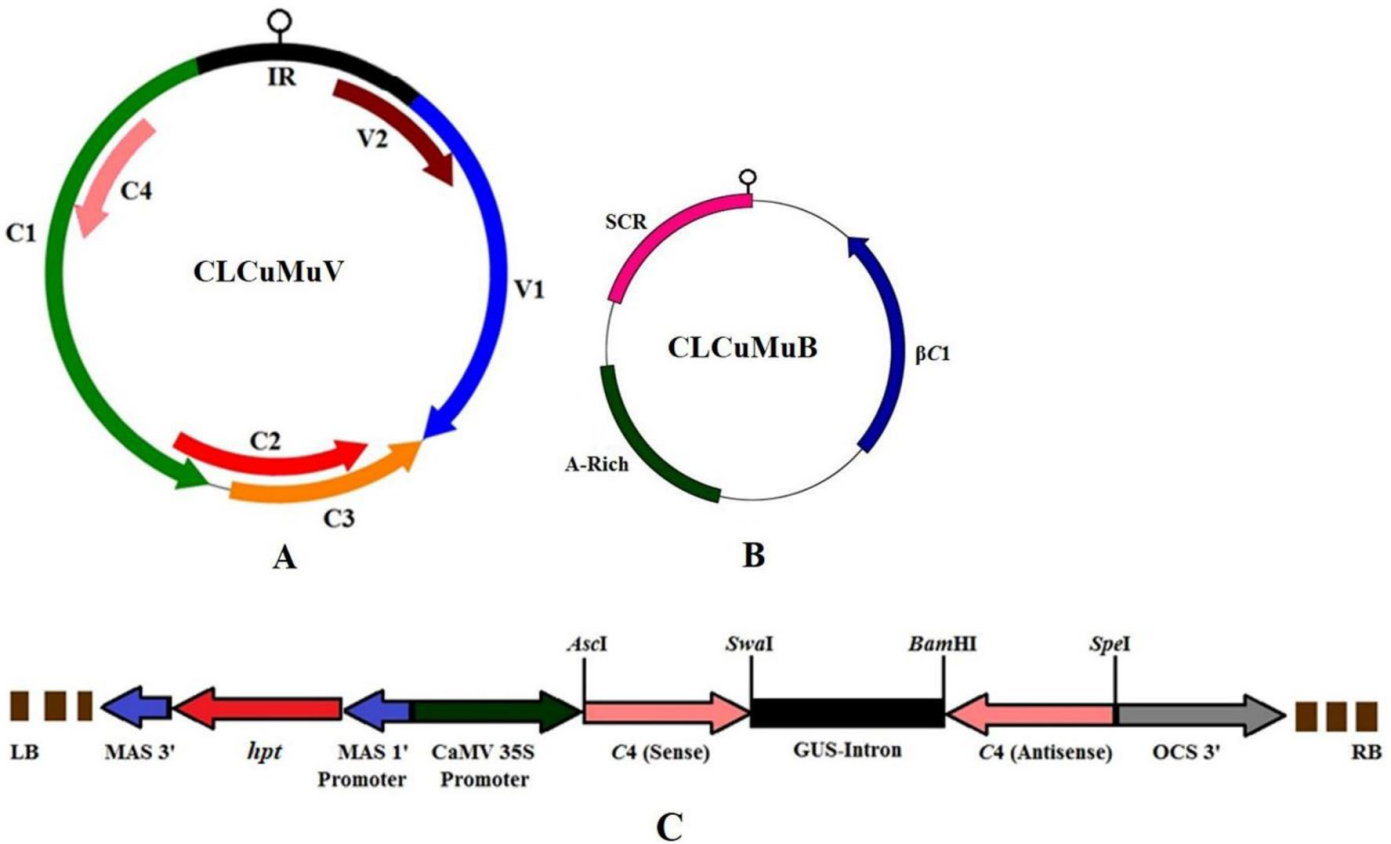
Genome organization of *Cotton leaf curl Multan virus* (CLCuMuV) (**A**) and associated Cotton leaf curl Multan betasatellite (CLCuMuB) (**B**). (**A**) Genome representing complementary-sense genes *C1*, *C2*, *C3*, *C4* (silencing suppressor) and virion sense *V1*, *V2* genes. (**B**) Betasatellite β*C1* gene encoding C1 protein. Abbreviations: A rich-Adenine-rich region; IR-Intergenic region; SCR-satellite conserved region. (**C**) Development of *Cotton leaf curl Multan virus*-*C4* intron-hairpin RNAi construct. The linear T-DNA map of intron-hairpin (ihp) RNAi construct in binary expression vector pFG*C*1008, in sense and antisense orientation separated by GUS intron. The complete RNAi cassette was under regulatory control of CaMV 35S promoter and OCS terminator, and having *hpt* as a plant selectable marker. Abbreviations: CaMV 35S—*Cauliflower mosaic virus* 35S promoter, GUS intron—β-glucuronidase intron, OCS—Octopine synthase terminator, MAS I promoter—Mannopine synthetase I promoter, and *hpt*—*hygromycin phosphotransferase*.

The CLCuMuV genome is monopartite, circular, ss DNA with approx. 2.7 kb in size (Fig. 1A). It is transcribed bidirectionally in virion sense and complimentary-sense orientations via intergenic region (IR) present between the first ORF of the virion sense and complimentary-sense strand. DNA elements required for replication and transcription are present in the IR. While *V1* and *V2* genes are transcribed from the virion sense, *C1*, *C2*, *C3*, *C4* and *C5* genes from the complimentary-sense strand^8^. The *C1* encodes the replication associated protein (Rep) that participates in rolling circle replication and regulation of complimentary sense gene. Transcriptional activator protein, expressed by the *C2* gene, regulates transcription of coat protein and movement protein encoding genes. Furthermore, it is involved in gene silencing. *C3* encodes the replication enhancer protein that leads to accumulation of viral DNA inducing symptom development and assists to enhance Rep-mediated ATPase activity. The *C4* gene encodes a multifunctional protein which is involved in symptom determination, systemic movement of virus and suppressing RNA silencing synergistically with transcriptional activator protein. In *East African cassava mosaic virus* and *African cassava mosaic virus*, AC4 protein has been shown to suppress gene silencing following binding of siRNA^9^. A comparative study on silencing suppressor genes of CLCuMuV demonstrated that the C4 protein may prevent siRNA from binding to RNA induced silencing complex (RISC) both prior to duplex binding as well as dicer mediated cleavage of long dsRNA^10^. Though C5 protein may participates in replication of DNA, it remains insignificant in viral infection. The CP (V1), which is multifunctional, is involved in genome packaging, insect-mediated transmission and spread of the virus. The *V2* gene expresses pre-coat protein that interacts with silencing suppressor genes such as *C2* and *C4*.

The begomoviruses are associated with three satellite molecules namely alphasatellite, betasatellite and newly-characterized deltasatellite^4,11^. These satellites depend on their helper virus genome for encapsidation and systemic infection. Alphasatellite encodes a single Rep protein which is non-pathogenic and enhances capability of autonomous replication in host plant^12^. While several distinct alphasatellites have been identified in CLCuD—affected cotton, they have not been shown essential either for development of the disease or virus/betasatellite activity. The betasatellites, ssDNA molecules, do not reveal any sequence homology with their helper viruses except stem loop structure having nonanucleotide (TAATATTAC) sequence^13,14^. The stem loop structure structure is present within highly conserve sequence called as satellite conserved region. Furthermore, adenine rich sequence and a single coding sequence (βC1) is located in the complimentary sense of the betsatellites (Fig. 1B). Betasatellite encodes a single βC1 protein which is a pathogenicity determinant, suppressor of host defence and involved in upregulating the level of viral DNA in planta^10,15,16^. Deltasatellites, small (~ 675 nts) non-coding satellites, are rarely encountered and not associated with CLCuD. Similar to C4 protein, βC1 acts as a suppressor of RNA silencing following binding to host’s DICER-like proteins required for biogenesis of small RNA molecules namely small interfering RNA (siRNA) and microRNA (miRNA)^17^.

Efforts are being made to develop cotton variety resistant to CLCuD. Control of CLCuD through resistance breeding has not been promising due to availability of limited resistance source and frequent emergence of recombinant begomoviruses^18^. RNAi based approaches have shown remarkable success against begomovirus infection following homology-based degradation of virus gene and its transcript RNA through transcriptional gene silencing (TGS) and post-transcriptional gene silencing (PTGS) mechanism in host plants^19–21^. RNAi is a highly conserved mechanism known to control gene expression in sequence specific manner by short non-coding RNAs (siRNA, miRNA) molecules^22–24^. These non-coding RNAs are closely related in their biogenesis and mode of action^17^. siRNA is originated from long double-stranded RNA (dsRNA) which is further processed into short (21–25 nts) single-stranded mature siRNA. Apart from regulating gene expression, siRNA is implicated in a variety of processes, such as defence against viruses, establishment of hetero-chromatin formation, DNA methylation, silencing of transgenes and post-transcriptional regulation of mRNA^25,26^. Interestingly, siRNA target the genes from which they are derived and repress any target mRNA showing substantial sequence complementarity.

In begomoviruses, both TGS and PTGS, involve processing of dsRNA by Dicer-like protein into siRNA which are subsequently incorporated into an enzyme complex termed as RISC. The RISC guides its argonaute protein to degrade target mRNA homologous to the siRNA in sequence specific manner. RNAi based antiviral defense mechanism in plants is designed following transgene expression of a virus specific dsRNA cognate to viral genome. A potential RNAi triggers molecules which can be successfully transformed into plants via expression of intron hairpin (ihp) construct carrying viral genome sequence into siRNA. In this study, *G. hirsutum* cv. HS6 was genetically transformed with the ihpRNAi construct harbouring the silencing suppressor gene (*C4*) of CLCuMuV to impart CLCuD resistance. The transgenic cotton lines, thus produced, were evaluated for resistance to CLCuMuV/CLCuMuB infection.

## Results

### Transformation of *G. hirsutum* cv. HS6

A total of approx. 1000 embryo apices co-cultivated with *Agrobacterium tumefaciens* GV3101 led to the production of nine transgenic (T_0_) lines (namely T_0_.3, T_0_.4, T_0_.6, T_0_.8, T_0_.10, T_0_.25, T_0_.26, T_0_.27 and T_0_.28 ) (Suppl Fig. SF1).

### Production of T_1_ progeny

Seeds (10–12 per line) of *G. hirsutum* cv. HS6 (T_0_) lines were allowed to germinate on MS selection media supplemented with hygromycin. The germinated (considered as CLCuMuV-resistant) and non-germinated (supposedly CLCuMuV-sensitive) seeds, maintained a germination ratio of 3:1 against hygromycin resistance on the selection medium. The presence of transgene in the T_1_ plants was confirmed in a total of 43 plants reflecting ~ 72% positive. This ratio was consistently observed in most of the lines (Supplementary Table ST2), and may be correlated with the Mendelian segregation (3:1) of a single dominant gene^27^.

### Molecular characterization of transgenic lines

#### Detection of *hpt* and transgene (*C4*) insert

**Figure 2:**
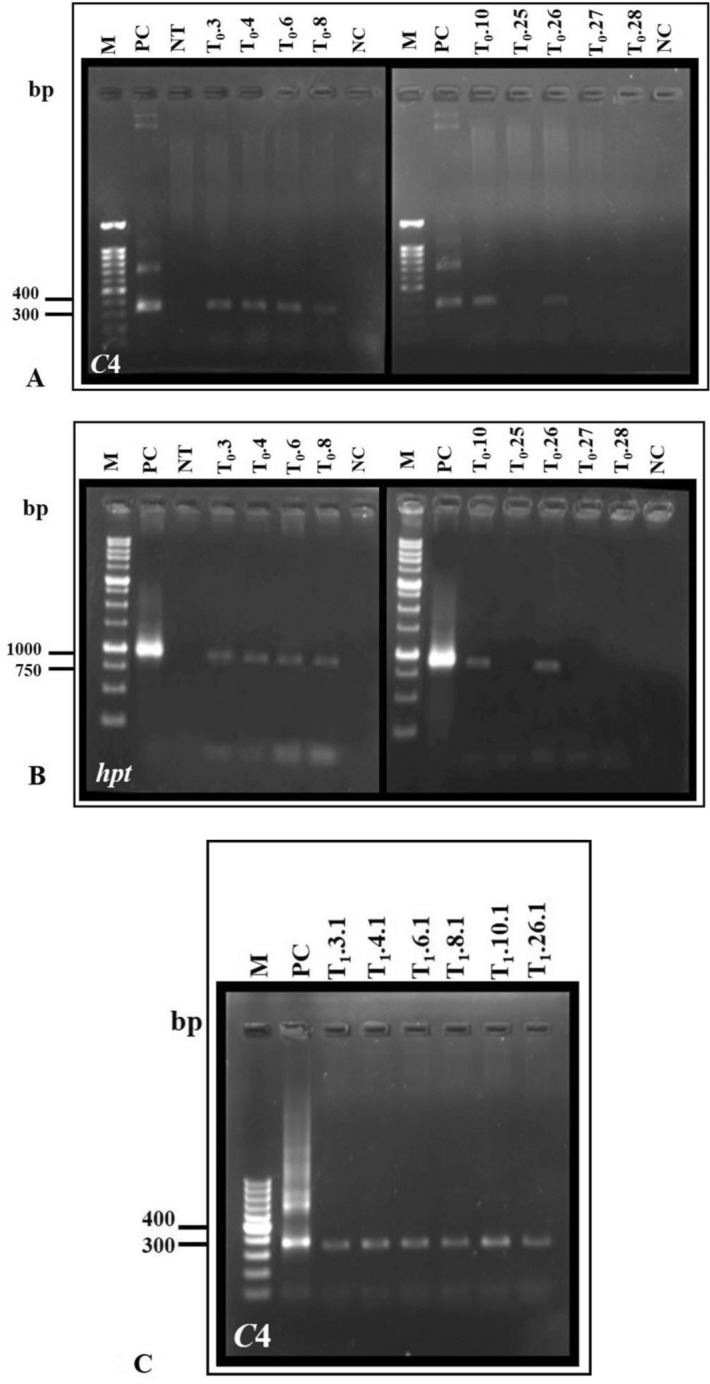
PCR amplification of: (**A**) silencing suppressor *Cotton leaf curl Multan virus* CLCuMuV-*C4* gene, (**B**) *hygromycin phosphotransferase* (*hpt*) gene in transformed T_0_ *G. hirsutum* cv. HS6 plants. T_0_.3, T_0_.4, T_0_.6, T_0_.8, T_0_.10 and T_0_.26 denote PCR positive lines, while T_0_.25, T_0_.27 T_0_.28 were found PCR negative. M is 100 bp DNA marker, PC is positive control, NT is non-transformed control and NC is negative controls. (**C**) CLCuMuV-*C4* gene in T_1_ *G. hirsutum* cv. HS6- T_1_.3, T_1_.4, T_1_.6, T_1_.8, T_1_.10 and T_1_.26 lines employing *C4* gene specific primers. M is100 bp DNA marker, In PC, construct plasmid DNA served as the template; In NC/NT, genomic DNA from non-transformed control was employed as the template in PCR.

The integration of transgenes (CLCuMuV-*C4*, *hpt*) in the nine putative transformed (T_0_) plants was checked by PCR. Among them, six plants (T_0_.3, T_0_.4, T_0_.6, T_0_.8, T_0_.10 and T_0_.26) were found PCR positive carrying copies of *C4* and *hpt* genes (Fig. 2A, B).

Similarly, PCR was performed to confirm the presence of the transgene (*C4*) in the transgenic T_1_ lines. All the six lines were found PCR positive for the *C4* gene (Fig. 2C).

### Southern hybridization

Southern blot hybridization using a DNA probe specific to the *C4* gene was performed. The transgene integrated in T_0_ lines was detected at a single locus in the lines T_0_.6, and T_0_.26, while double loci integration event was observed in the T_0_.3, T_0_.4, T_0_.8 and T_0_.10 lines (Suppl Fig. SF2A).

Analysis of the T_1_ lines also showed low copies (2–3) of transgene insertions. The T_1_ lines, namely, T_1_.3.1, T_1_.6.1, T_1_.8.1 and T_1_.26.1 showed two copies of the transgene integration events, while T_1_.4.1and T1.10.1 carried three integration events (Suppl Fig. SF2B).

### Semi-quantitative RT-PCR analysis

Different level of dsRNA expression was observed in all the six transgenic (T_0_) lines. While, transcript level appeared to be higher in T_0_.8, relatively a lower expression was exhibited in T_0_.10 and T_0_.26 transgenic lines.

Likewise, in the T_1_ lines, intensity of the transcript level appeared to be higher in the line T_1_.8.1. In contrast, lines T_1_.10.1 and T_1_.26.1 exhibited lower intensity bands among all the T_1_ lines. An intensity of relatively intermediate level was observed in T_1_.3.1, T_1_.4.1 and T_1_.6.1 lines. The expression of the transgene was found to be comparable with that of internal control *GAPDH* gene, yielding more or less of equal intensity amplicons.

### qRT-PCR

The Quantitative Real-time data (Ct values) revealed that ihpRNA-C4 was transcribed into dsRNA in all the transgenic (T_0_ and T_1_) lines. Taking *GAPDH* gene as an internal control, the RQ value of the dsRNA was found to be higher in T_0_ as compared to control plant. T_0._8 being the highest, the lowest transcription was observed in T_0_.26 (Suppl Fig. SF3A).

The RQ value of the dsRNA transcripts was also exhibited high in T_1_ transgenic lines as compared to NT (non transformed) control plants (Suppl Fig. 3B). Transgenic lines T_1_.8.1, T_1_.6.1,T_1_.10.1, T_1_.4.1, and T_1_.3.1 exhibited 12, 12, 12, 11 and 11-fold, increase respectively, while T_1_.26.1 showed lowest expression (nine-fold) as compared to the NT control plants (Suppl Fig. SF3B).

### Northern hybridization

**Figure 3:**
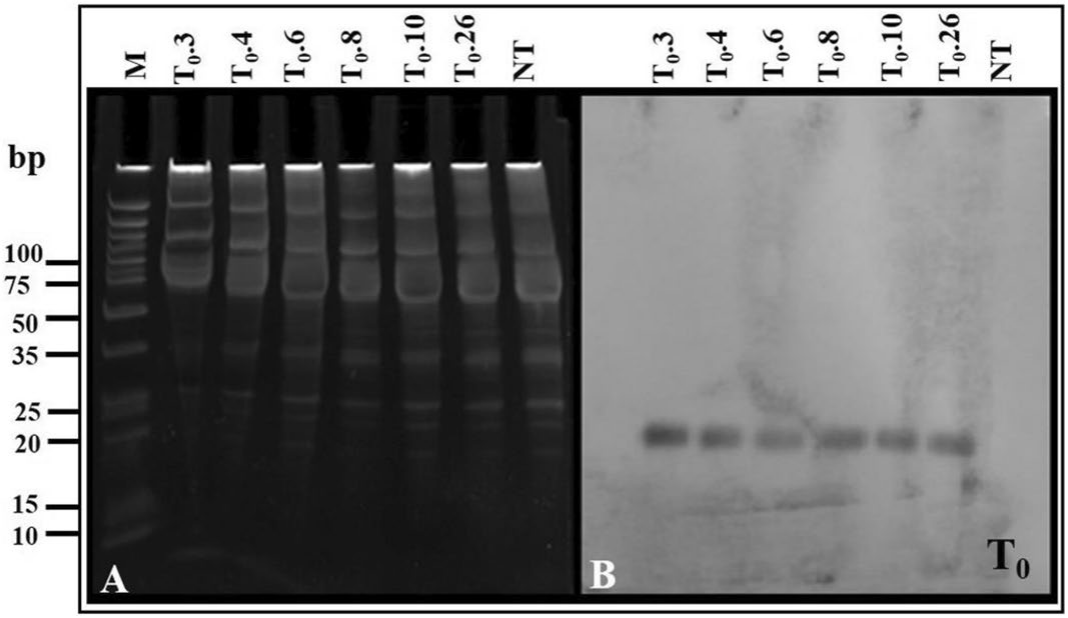
(**A**) Detection of small RNAs population of transgenic  (T_0_) *G. hirsutum* cv. HS6- T_0_.3, T_0_.4, T_0_.6, T_0_.8, T_0_.10 and T_0_.26 and non-transformed (NT) control resolved through denaturing polyacrylamide gel electrophoresis. (**B**) Northern blot analysis of siRNAs generated in transgenic T_0_ lines transferred onto HybondN membrane and hybridized with DIG-11-dUTP-labelled *C4*-derived probe.

It was performed to check the expression of siRNA levels in the transgenic lines. The *C4* gene-based DIG-11-dUTP-labeled probe yielded positive signals corresponding to the 20–25 nt region of ultra-low range marker, indicating the processing of ihpRNAi-*C4* construct subsequently producing siRNAs in the transgenic lines. The intensity of siRNA bands appeared to be slightly higher in T_0_.3, T_0_.4, T_0_.8, T_0_.10 and T_0_.26 lines as compared to the line T_0_.6 (Fig. 3A, B).

Intensity of siRNA signals seemed to be of equal intensity in all the T_1_ lines, except T_1_.3 which was slightly higher (Suppl Fig. SF4A, B).

### Whitefly-mediated virus inoculation of CLCuMuV and assessment of CLCuD symptoms

**Figure 4:**
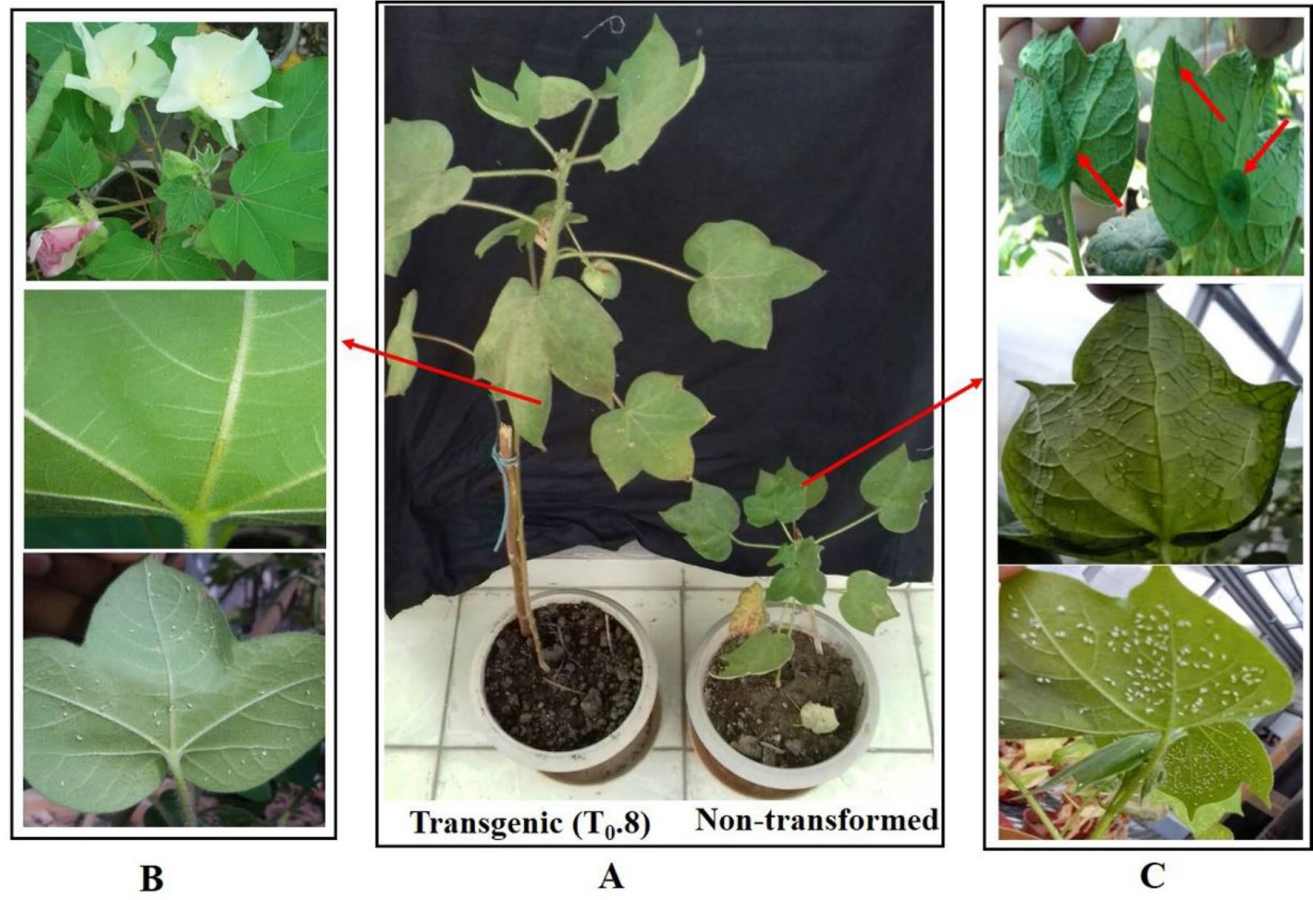
(**A**) Assessment of cotton leaf curl disease (CLCuD) symptoms in transgenic *G. hirsutum* cv. HS6 (T_0._8) and non-transformed control plants after inoculation with viruliferous whiteflies (*Bemisia tabaci*) under greenhouse conditions. (**B**) No characteristic CLCuD symptoms developed on the leaves of transgenic lines 60 days post inoculation and transgenic plant showed normal phenotype. (**C**) CLCuD symptoms were developed on non-transformed control *G. hirsutum* var. HS6 plant such as vein thickening, leaf curling and enations were observed.

Following CLCuMuV/CLCuMB inoculation of transgenic lines with viruliferous whiteflies, symptom expression were periodically evaluated over a period of two months under strict greenhouse conditions (Fig. 4A–C). There were no visible CLCuD symptoms in the transgenic lines as compared to control showing severe symptoms (Fig. 4A, B). The initial symptoms of CLCuD appeared in the non-transformed control plant at 15 dpi. While the transgenic plants were apparently seen healthy without visible symptoms at 30 dpi when the characteristic symptoms were fully expressed in the control plants. The symptoms consisted of vein thickening, leaf curling, leafy enations and stunting of plants with no or few ball settings as observed in the control plants (Fig. 4A, C). Contrary, all the transgenic lines (T_0_ and T_1_) remained apparently symptom free maintaining their natural vegetative and reproductive growth (Suppl Fig. SF8).

Remarkably, 8-weeks post-inoculation none of the transgenic lines exhibited any CLCuD symptoms. Apparently, no phenotypic differences were observed among the transgenic and NT control plants, except appearance of severe symptoms in the control plants.

### Detection of CLCuMuV and betasatellite DNA

PCR failed to detect CLCuMuV and CLCuMuB in T_0_ lines eight weeks post-inoculation. While, the control plants yielded DNA fragments corresponding to the *CP* gene (~ 780 bp) and betasatellite (~ 1.3 kb) (Suppl Fig. SF5A, B).

Similarly, PCR did not lead to amplification of CLCuMV and betasatellite in (T_1_) lines at eight weeks post-inoculation. However, non-transformed control plants yielded DNA fragments of CLCuMuV and CLCuMuB (Suppl Fig. SF6A, B).

### Rolling circle amplification

Total genomic DNA isolated from the transgenic lines and NT control plants was subjected to Rolling Circle Amplification (RCA). The RCA products served as templates for PCR amplification of the virus and betasatellite using primers specific to the *CP* gene and betasatellite as described in above section. RCA products, derived from the T_0_ lines at 15 dpi, did not detect the virus and betasatellite, though it tested positive in control plants (Suppl Fig. SF7). Thus, clearly demonstrating that there was no detection of virus/betasatellite DNA in any of transgenic (T_0_) lines.

However, at 30 dpi, very faint DNA fragments of CLCuMuV and betasatellite were observed in lines T_0_.10 and T_0_.26 (Suppl Fig. SF7A, B).RCA analysis at 60 dpi, revealed higher intensity DNA fragments CLCuMuV/betasatellite in control plants, as compared to all other T_0_ transgenic plants (Suppl Fig. SF7A, B).

**Figure 5:**
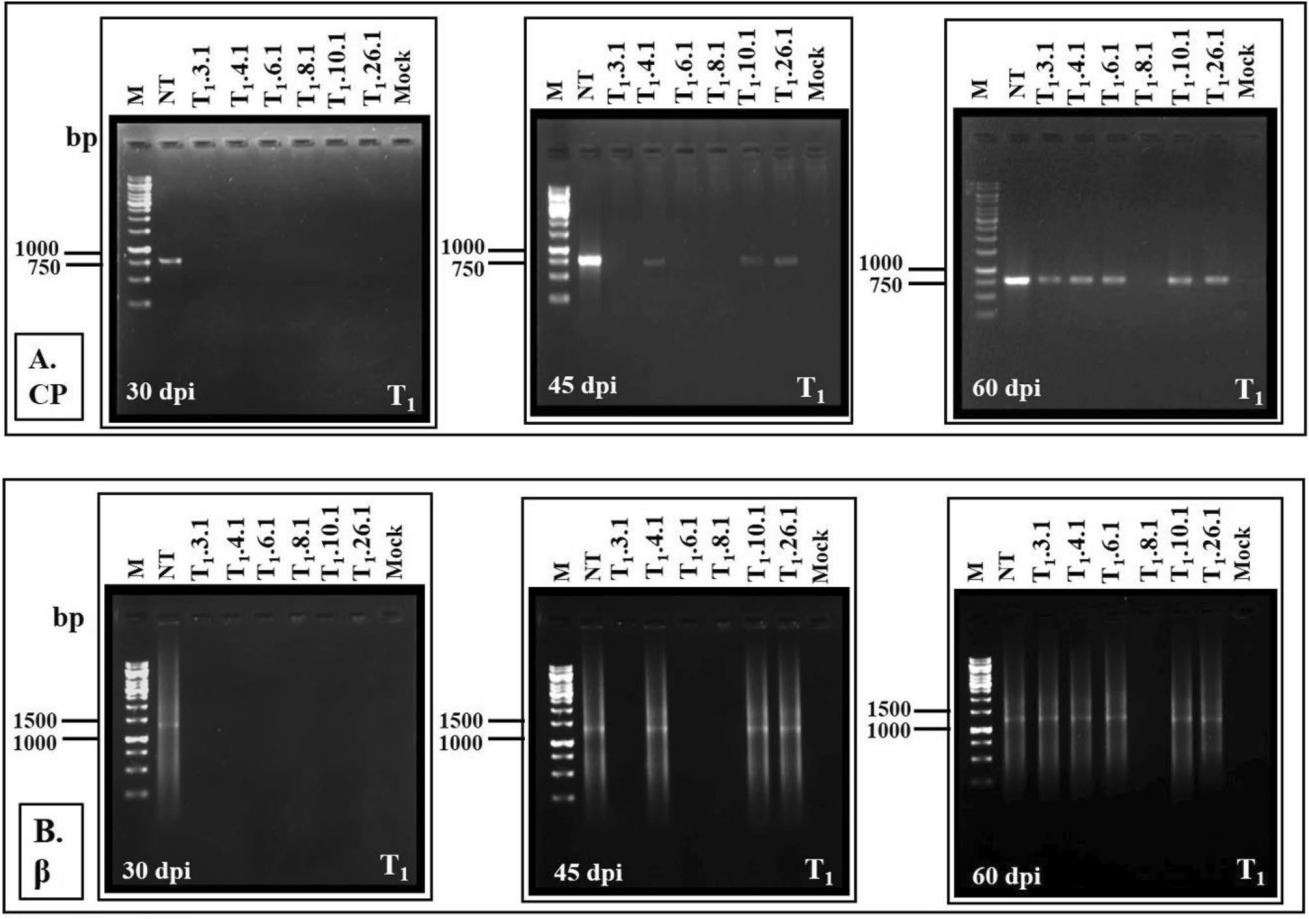
RCA-PCR based detection of: (**A**) CLCuMuV-coat protein (CP) gene, and (**B**) CLCuMuB. Total genomic DNA of T_1_ progeny *G. hirsutum* cv. HS6- T_1_.3, T_1_.4, T_1_.6, T_1_.8, T_1_.10 and T_1_.26 plants and non-transformed control plants were isolated at 30, 45 and 60 dpi with viruliferous whitefly, and RCA was performed. The RCA product served as a template in PCR employing primers specific to CLCuMuV-CP and betasatellite. Amplification of the CP gene (~ 780 bp) and the CLCuMuB (~ 1.3 kb) was detected in non-transformed control plants only at 30 dpi. A low degree amplification of CP gene and betasatellite was recorded in T_1_.4, T_1_.10 and T_1_.26 lines at 45 dpi and in transgenic HS6- T_1_.3, T_1_.4, T_1_.6, T_1_.10 and T_1_.26 lines at 60 dpi. M is 1 kb DNA marker, NT is non-transformed control and Mock is uninoculated control.

The progression of CLCuMuV/CLCuMuB in the T_1_ lines was also assayed by RCA. No virus/betasatellite DNA fragments were identified at 30 dpi, while they could be detected in the NT control plants. In lines (T_1_.4.1, T_1_.10.1 and T_1_.26.1), CLCuMuV/betasatellite could be detected at 45 dpi (Fig. 5A, B).

However, a low accumulation of the virus and betasatellite DNA was detected in all the lines except in T_﻿1_.8.1 at 60 dpi (Fig. 5A, B).

## Discussion

In this study, ihpRNAi construct carrying the *C4* gene of CLCuMuV was evaluated for its efficacy in combating the CLCuD infection following transformation in *G. hirsutum* cv. HS6 plants. The expression of ihp dsRNA-C4 -derived siRNA, assessment of CLCuD resistance and progression of the CLCuMV/CLCuMB levels in transgenic cotton were studied. The expression of siRNA was high in the transgenic lines exhibiting no visible CLCuD symptoms. They showed resistance against CLCuD following viruliferous whitefly-mediated inoculation. All the transgenic lines exhibited  significantly lower level of virus as well as associated betasatellite in comparison to the control plants. A low number of transgene integration was observed in transgenic lines. In contrast to T_0_ lines, a slight variation in the number of transgene integration was evident in the T_1_ lines which could be attributed to minor variations in the genetic background or differences in the plant microenvironments^27^. Insertion of multiple copy transgene in the plant genome favours resistance leading to higher production of transgene derived siRNA generation.

qRT-PCR analysis showed that the level of ihp dsRNA was unexpectedly high in the transgenic lines, displaying high level of siRNA expression. A high level of dsRNA could be due to its incomplete processing^28^.

A significant lower level of the virus/betasatellite DNA as compared to the control plants was attributed to the silencing of CLCuMuV-*C4* gene by hpRNAi-derived siRNA. It may be mentioned that the *C4* gene is fully contained inside the *C1* gene in a different ORF. The *C1* gene encodes Rep protein which is absolutely required for virus replication. Thus, additional targeting of *C1* transcript by the *C4*-derived siRNA may not be overlooked. That may explain the detection of a low virus titer.

The *C4* and β*C1* genes appear to be important targets for the control of the begomovirus infections as they are strong RNA silencing suppressors and play a vital role in the survival of viruses. *AC4*/*C4* gene encodes a multifunctional protein which acts as pathogenicity determinants in monopartite and bipartite geminiviruses. In a previous study, role of individual genes encoded by four begomoviruses (mono/bipartite) on infection cycle and level of miRNAs involved in plant development, was investigated. A disruption of the *C4* gene (monopartite begomovirus) resulted in attenuation of symptoms and low infectivity. The C4 protein was responsible to change the levels of miRNAs and was probably involved in modulating gene expression to create an environment favourable for virus proliferation^29^. In CLCuKoV, it is involved in virus spread and in modulating symptoms, but dispensible for maintenance of the betasatellite suggesting a lack of suppressor activity. If present, does not overcome the silencing based resistance to virus movement^30^.

In a first report of a begomovirus requiring four suppressor genes to establish infections under field conditions, efficacy and relative strength of CLCuMuV and CLCuMB-encoded suppressors to suppress RNAi silencing were compared. While C4 protein exhibited silencing suppressor activity, V2 being the strongest. It could bind both to long dsRNA as well as siRNA^31^. In another report, CLCuKoV C4 protein was demonstrated to prevent the spread of systemic silencing. It could bind to long dsRNA and both long ds/ssDNA, but not to sRNA/DNA^32^.

In the present study, transgenic *G. hirsutum* lines did not display any symptoms at two months post-inoculation of CLCuMuV/CLCuMB. While conventional PCR failed to detect virus DNA and betasatellite in any transgenic line at 60 dpi, RCA-PCR was positive in all except one. However, level of the virus accumulation was much lower in comparison to non-transformed plants, and the plants displayed no characteristic CLCuD symptoms.

*G. hirsutum* plants following transformation led to generation of siRNA responsible for suppressing CLCuD symptoms, though  CLCuMuV/betasatellite could be detected at a later stage. This type of observed resistance may be termed as tolerance, where a balance between the host defence response and virus counter defence seems to exist that allows the virus to survive in the host without causing apparent symptoms. These results are consistent with earlier studies where RNAi has imparted tolerance^33^. In a previous study, intergenic region (CLCuMuV) carrying ihpRNAi construct was transformed in Indian cotton cv. ‘Narsimha’. Transformed (T_0_) cotton plants did not develop symptoms at 90 dpi. However, second (T_1_) generation was not assessed. A tolerance phenomenon was observed as some of the transformed plants revealed the presence of virus^34^. In a preliminary study, tobacco plants transformed with βC1 exhibited no or delayed CLCuD symptoms following CLCuMuV/betasatellite inoculation. Titre of the virus or presence of βC1-derived siRNA could not be tested in the transgenic lines^35^.

In the absence of an immune cultivar, availability of virus/betasatellite tolerant variety may be an alternative to minimise CLCuD losses. In this study, cotton lines showing RNAi-mediated tolerance resistance against CLCuD infection have not been tested in fields. Presence of virus in the tolerant plants, and the variation in virus complex causing CLCuD, however, may pose a risk development of recombinant virus mutants that may produce novel viruses capable of overcoming resistance and spread to other crop plants^36,37^. Reference may be made of cassava mosaic disease in which interaction between two distinct begomoviruses (*African cassava mosaic virus-*CM and *East African cassava mosaic virus*) are responsible for developing severe disease symptoms. The synergism is due to the action of silencing suppressor AC2 and AC4 genes that target different steps in RNA silencing and overcome host defense^38^.

## Conclusion

In this study, transgenic *G. hirsutum* cv. HS6 lines significantly reduced the virus DNA accumulation and attenuated CLCuD symptoms following CLCuMuV-*C4* derived RNAi strategy. Further, siRNAs were triggered at a high level and symptoms remained escaped. However at the later stage, the transgenic plants were mildly infected, supporting CLCuMuV/CLCuMB replication. Role of *C4* gene-derived siRNA in developing virus resistance was investigated for the first time in transgenic *G. hirsutum* cultivar against CLCuD infection**.**

## Materials and methods

### ihpRNAi construct preparation

ihpRNAi construct harbouring *C4* gene sequence in sense-antisense orientation was produced in binary vector pFG*C*1008. Full-length clone of *Cotton leaf curl Multan virus-Raj* (CLCuMuV-Raj, Accession No. GQ220850) was used as a template for PCR-based amplification of *C4* gene followed by its cloning in binary vector pFG*C*1008 in sense orientation at *Asc*I and *Swa*I, and in antisense orientation at *Bam*HI and *Spe*I restriction sites (Sambrook and Russell 2012). The orientations of the gene were separated by a GUS-intron under the transcriptional control of constitutive CaMV 35S promoter containing *hpt* gene as a plant selection marker (Fig. 1C). The developed ihpRNAi cassette was mobilized into *Agrobacterium tumefaciens* strain GV3101 using freeze thaw method. The integrity of the construct was tested by sequencing (Xcelris genomics, India).

### Transformation of *Gossypium hirsutum* cv. HS6

The young embryo apex explants excised from germinating seeds of *G. hirsutum* cv. HS6 were, were transformed with *A. tumefaciens* strain GV3101 harbouring ihpRNAi-C4 construct. Cotton transformation was done as described earlier^39,40^. A total of 1000 number of explants (in different batches) were transferred on the solid MS basal medium supplemented with cefotaxime (250 mg/L) (Suppl Fig. SF1A). The explants were placed on selection medium supplemented with hygromycin (50 mg/L) and cefotaxime (250 mg/L) (B), sub-cultured on shooting medium for 3 weeks (C). The regenerated shoots were subsequently shifted on root induction medium (D, E). For 2–3 weeks, plantlets with well-developed roots were placed in Hoagland medium (F). The hardened plants were transferred to pots in greenhouse under controlled conditions (G, H).

### Collection and germination of seeds of transgenic T_0_ lines

The transformed (T_0_) *G. hirsutum* cv. HS6 plants were self-pollinated under strict greenhouse conditions for the production of seeds. Second generation transgenic (T_1_) plants were raised following collection and subsequent germination of transgenic (T_0_) plants seeds. The 10–12 seeds of each (T_0_) transformed line were germinated on MS selection medium containing hygromycin (50 mg/L), cefotaxime (250 mg/L) and BAP (2.0 mg/L).

### Confirmation of transformed *G. hirsutum* cv. HS6 plants

Total genomic DNA from leaves of putative transformed (T_0_) *G. hirsutum* cv. HS6 plants was isolated using DNeasy plant mini kit according to manufacturer’s instructions (Qiagen, Germany). The integration of transgenes in putative T_0_ transformed plants was confirmed by PCR and Southern hybridization.

### PCR

PCR was performed employing *C4* gene specific (*C4* For/Rev primers, Supplementary Table ST1) using genomic DNAs (T_0_ plants) as templates. PCR mixture (200 ng of template DNA, 10X *Taq* buffer A, 2.5 mM each dNTP, 50 pmol of each primer, 3 U/μL *Taq* DNA polymerase) with parameters: initial denaturation at 95 °C for 7 min followed by 30 cycles of final denaturation at 95 °C for 50 s, annealing at 50 °C for 50 s, initial extension at 72 °C for 30 s and final extension of 72 °C for 5 min.

### Southern hybridization

For Southern hybridization, 10 µg of total genomic DNA isolated from the PCR positive transgenic (T_0,_ T_1_ plants) and non-transformed *G. hirsutum* cv. HS6 control plants was subjected to restriction digestion with 10U *BamH*I (Fermentas, USA). The digested genomic DNA was electrophoresed and transferred on Hybond-N nylon membrane (GE healthcare, UK)^41^. DIG labelled DNA probe representing *C4* gene was prepared using labelling kit (Roche Diagnostics, Germany). The membrane was hybridized with the DIG labelled probe and subjected to the development of colour following incubation in in a freshly prepared Colour-substrate solution Nitro blue tetrazolium/5-Bromo-4-chloro-3-indolyl phosphate (NBT/BCIP).

### Expression of transgene transcript

Total RNA was isolated from the transgenic and non-transformed plants using RNeasy Plant Mini kit (Qiagen, Germany). cDNA was synthesized with M-MuLV RT-PCR Kit (Merck, Germany). Expression of the transgene transcript was analysed through Reverse transcriptase (RT)-PCR and Real Time (qRT)-PCR.

#### RT-PCR

It was performed to compare relative expression of the transgene transcript level with that of *﻿GAPDH* gene in transgenic (T_0_ and T_1_) lines using Qiagen Long Range 2Step RT-PCR kit, Germany with oligo primers specific to the *C4* (RT-*C4* For/Rev) and *GAPDH* (RT-*GA* For/Rev) genes (Supplementary Table ST1). PCR mixture was subjected to PCR amplification with parameters: initial denaturation at 94 °C for 5 min, followed by 35 cycles of final denaturation at 94 °C for 45 s, annealing at 55 °C for 45 s and initial extension at 72 °C for 30 s and final extension at 72 °C for 3 min.

#### Quantitative real-time PCR

It was conducted to quantify the level of expression of ihpRNA dsRNA transcript, in comparison to that of *﻿GAPDH* gene in the transgenic lines and non-transformed control plants. The housekeeping gene *﻿GAPDH* served as an internal control. The mixture contained template cDNA (100 ng), 20 pmol of each (RT-*C4* For/Rev and RT-*GA* For/Rev (Supplementary Table ST1) and Real Time master mix including SYBR green dye (2X KAPA SYBR® FAST Universal and 50X ROX high) (KAPA Biosystems, USA). The qRT-PCR programming conditions were as follow: initial denaturation at 95 °C for 5 min, followed by 40 cycles of final denaturation at 95 °C for 30 s, annealing at 55 °C for 30 s and extension at 72 °C for 30 s. All the target samples, internal control and negative control were taken in triplicates and the experiment was conducted twice.

The qRT-PCR generated data was statistically analysed to calculate the relative quantification (RQ) of ihpRNA transcript level in the transgenic and NT control plants. The StepOnePlus Real-Time PCR System, having StepOne™ Software v2.3 was used for the calculation of threshold cycle (Ct) values of all the target samples (Applied Biosystems, USA). The RQ of the target samples was calculated by Livak’s 2^−ΔΔCt^ method using mean Ct values of the target and the internal control gene from triplicate samples^42^. Standard deviation (SD) and standard error (SE) were determined from the triplicate samples.

### Detection of siRNAs in transgenic lines

#### Northern hybridization

Total small RNA population was isolated from the leaves of transgenic and NT control plants^43^. It was performed to assess the expression level of siRNA in transgenic (T_0_, T_1_) plants with respect to corresponding non-transformed cotton plants. The low molecular weight RNA were separated on denaturing polyacrylamide gel (PAGE 15%) and transferred onto a Hybond-N nylon membrane (GE Healthcare, UK). Hybridization was performed with CLCuMuV-*C4* gene-based DIG-11-dUTP labelled probe at 42 °C in DIG Easy Hyb solution using DIG DNA labelling and detection kit (Roche Diagnostics, Germany). The membrane was equilibrated in Detection buffer containing NBT/BCIP-T under Gel Doc™ EZ System (BioRad, USA) using the Image Lab™ software Version 4.1. siRNA fragments (20–25 nt long) were visualized, which were compared with the original PAGE exhibiting ultra-low range RNA marker (Thermo Scientific, Germany).

### Screening and evaluation of transgenic lines for resistance to CLCuD

Transgenic (T_0_, T_1_) plants were inoculated (at 4–5 leaf stage) with CLCuMuV/CLCuMB through whitefly (*Bemisia tabaci*)^44^. Approximately, 100 viruliferous whiteflies were placed on each transgenic and non-transformed control plants for 72 h inside insect proof chamber under controlled conditions. The plants were periodically assessed for 60 dpi for the development of symptoms.

### Detection of CLCuMuV/CLCuMB DNA in the transgenic lines

The presence of virus DNA and betasatellite molecules in transgenic plants were assayed by PCR and RCA.

#### PCR

Total genomic DNA was isolated from the transgenic and non-transformed plants. It was subjected to PCR for the specific detection of the CP gene of CLCuMuV and betasatellite molecules employing primer pairs For/Rev and Beta For/Rev specific for the CP gene of CLCuMuV and betasatellite molecules, respectively (Supplementary Table ST1), as described in previous sections.

#### Rolling circle amplification based PCR

Total genomic DNA was subjected to RCA using TempliPhi DNA amplification kit (GE Healthcare, UK). The RCA products served as the templates for amplification of the virus DNA and betasatellite employing primers specific for the CLCuMuV-*CP* gene and betasatellite (Supplementary Table T1). The PCR parameters remained same as described above.

## Supplementary Information

Supplementary Information 1.
