# Supplementary material for: Engineering tolerance to CLCuD in transgenic Gossypium hirsutum cv. HS6 expressing Cotton leaf curl Multan virus-C4 intron hairpin
Source: Sci Rep. 2021 Jul 8;11:14172. doi: 10.1038/s41598-021-93502-3 (PMC8266814; doi:10.1038/s41598-021-93502-3)
Supplement: Supplementary file 1 — Supplementary Information 1. [file 41598_2021_93502_MOESM1_ESM.doc]

**Supplementary Figures and Tables**

**Engineering tolerance to CLCuD in transgenic *Gossypium hirsutum* cv. HS6 expressing *Cotton leaf curl Multan virus* - *C4* intron hairpin**

**Mirza S. Baig^1,2^, Sadia Akhtar^1^, Jawaid A. Khan^1^***

1Department of Biosciences, Jamia Millia Islamia (Central University), Jamia Nagar, New Delhi, 110025, India

^2^Department of Molecular Medicine, Jamia Hamdard, Hamdard Nagar, New Delhi, 110062, India

*correspondingauthor[jkhan1@jmi.ac.in](mailto:jkhan1@jmi.ac.in)


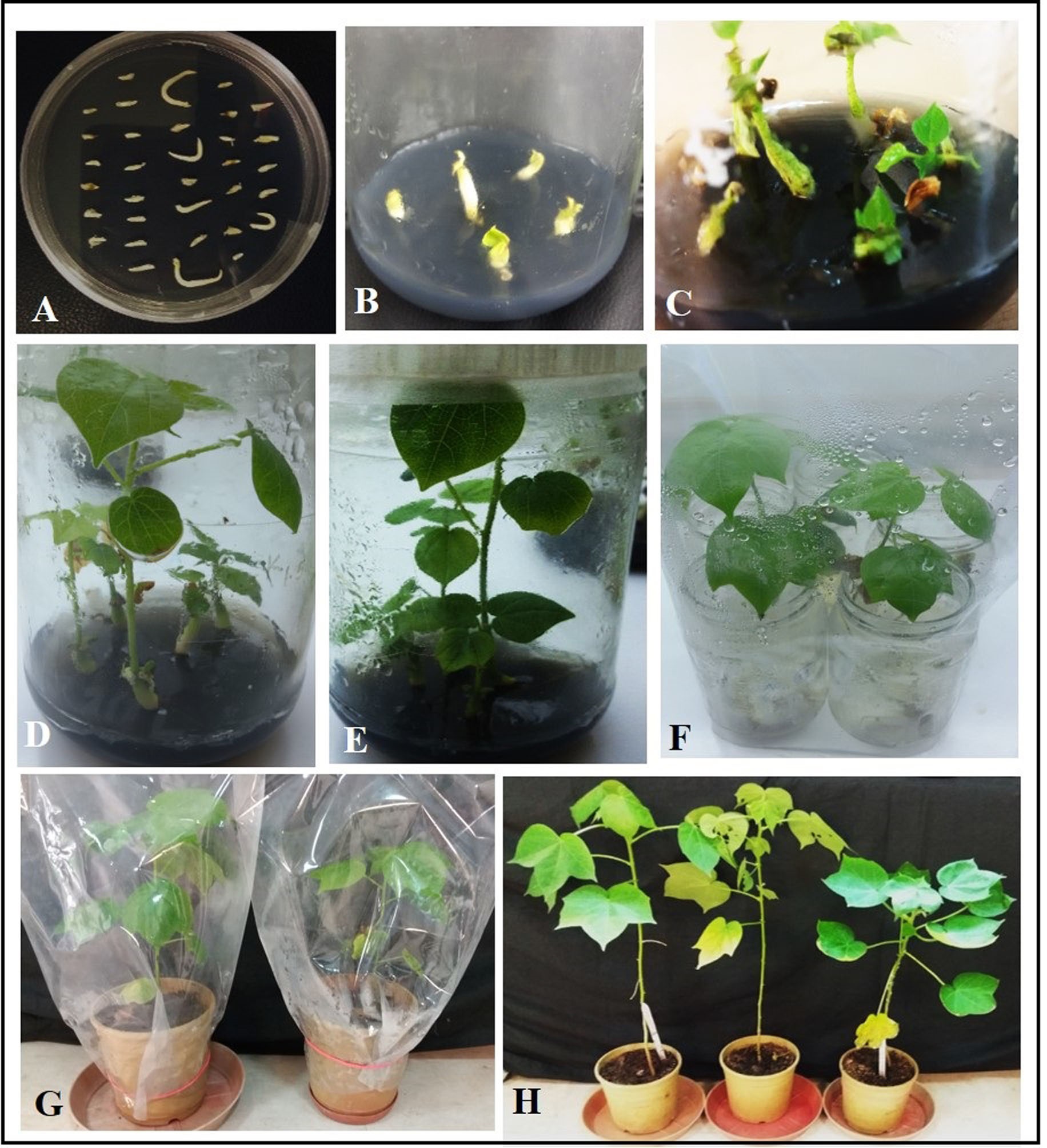


**Supplementary Figure SF1.** *Agrobacterium*-mediated transformation of *Gossypium hirsutum* cv. HS6 **(A)** co-cultivation of embryo apices, **(B)** embryo apices on selection medium, **(C)** regeneration of shoots on shoot induction medium, **(D)** Elongated shoots on shooting medium, **(E)** regenerated plantlet on rooting medium, **(F)** and in Hoagland medium, **(G)** plants in pots for acclimatization, and **(H)** transformed (T_0_) *G. hirsutum* cv. HS6 plants under greenhouse conditions.

**
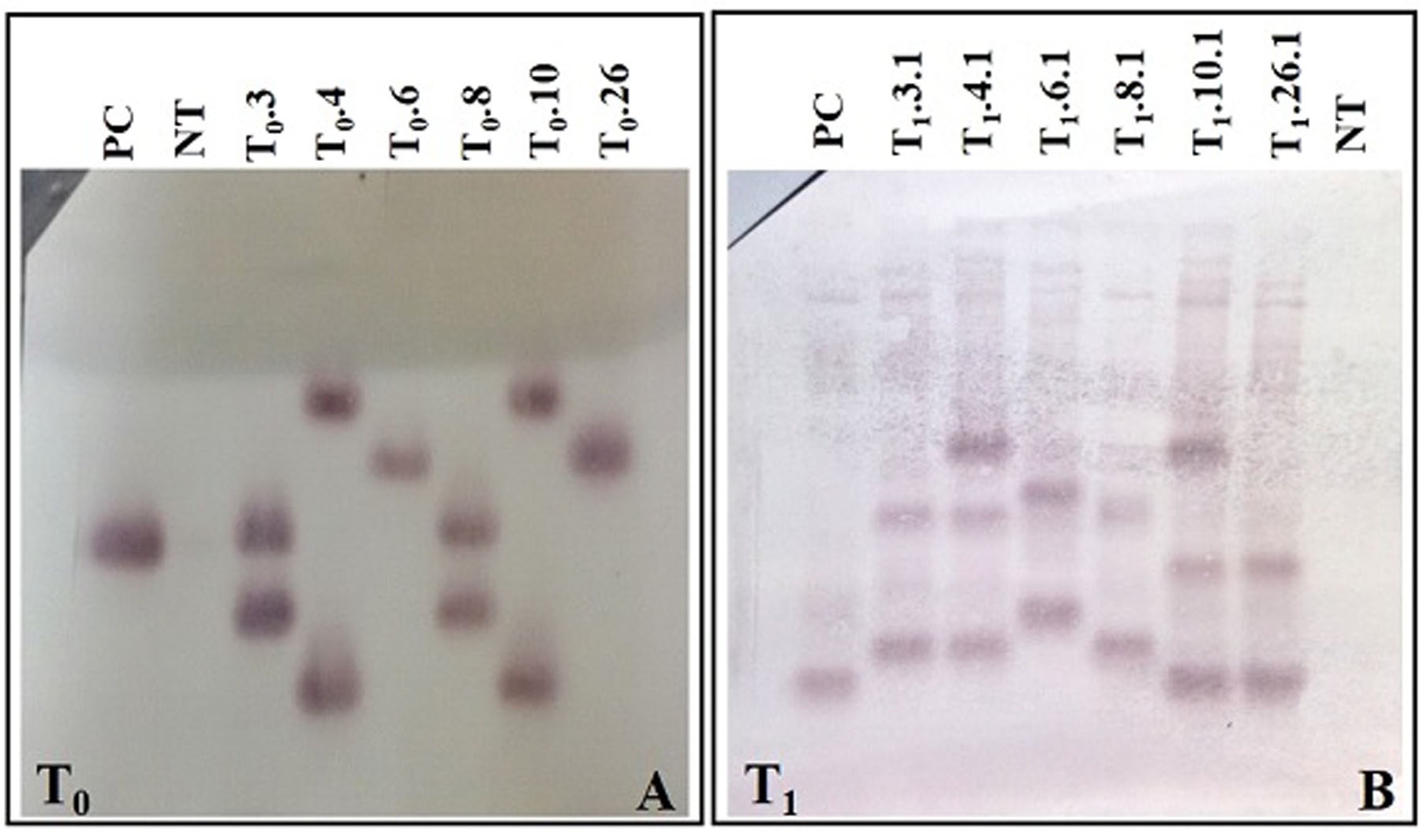
**

**Supplementary Figure SF2.** Southern blot analysis: **(A)** T_0_ *G. hirsutum* cv. HS6 lines T_0_.3, T_0_.4, T_0_.6, T_0_.8, T_0_.10, and T_0_.26; and **(B)** T_1_ lines T_1_.3.1, T_1_.4.1, T_1_.6.1, T_1_.8.1, T_1_.10.1, and T_1_.26.1. Hybridization was performed with CLCuMuV-*C4* gene-based DIG-11-dUTP-labelled probe. PC is positive control; NT is non-transformed control. Genomic DNAs of non-transformed and transformed *G. hirsutum* cv. HS6 plants were digested with *Bam*HI.


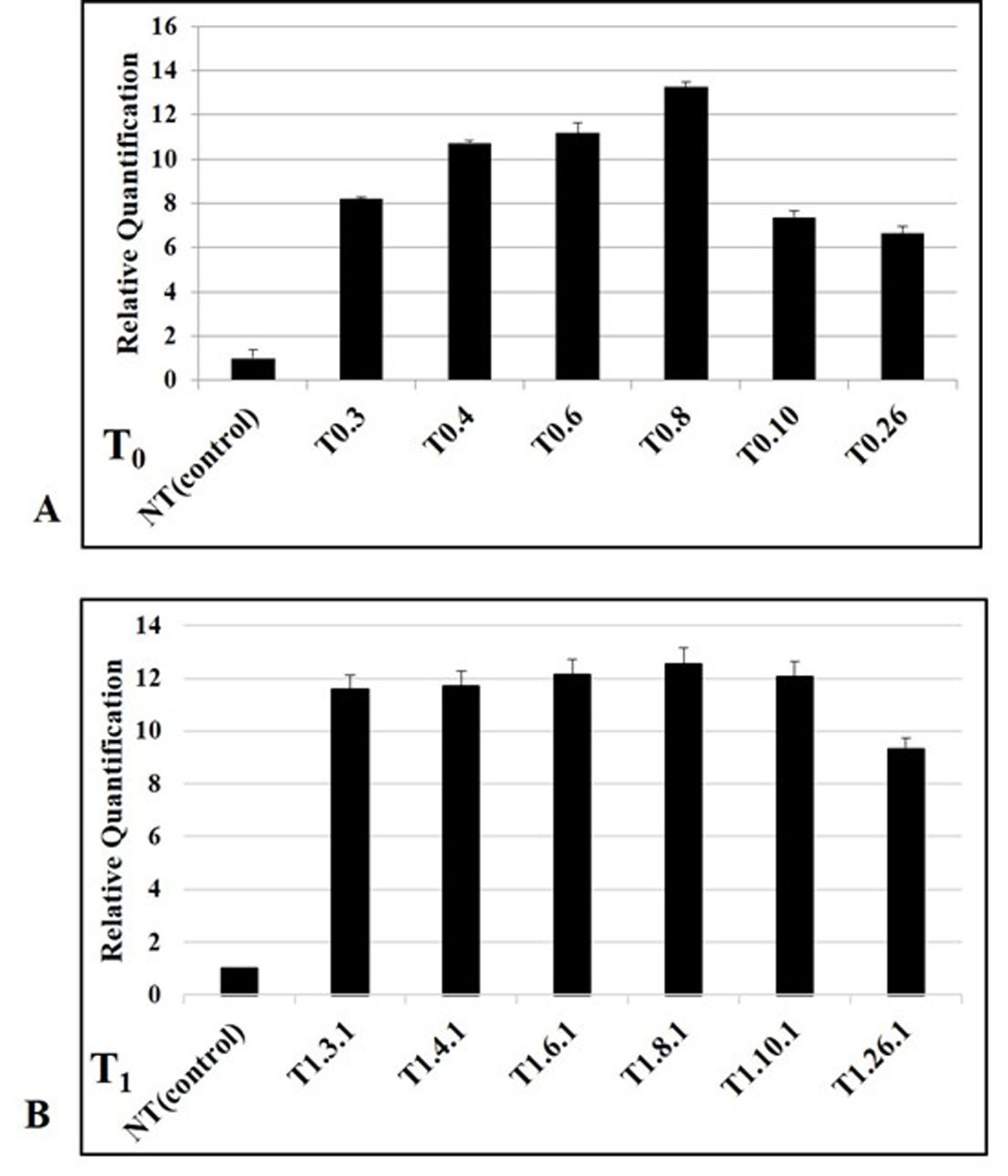


**Supplementary Figure SF3.** Quantitative Real-time (qRT)-PCR-based Relative Quantification (RQ) of the expression level of *C4* gene transcript in transgenic (T_0_ and T_1_) *G. hirsutum* cv. HS6 lines. cDNA was prepared from total RNA isolated from **(A)** transgenic (T_0_) *G. hirsutum* cv. HS6-L3, -L4, -L6, -L8, -L10, -L26 lines and, **(B)** transgenic (T_1_) *G. hirsutum* cv. HS6-L3(a), -L4(a), -L6(a), -L8(a), -L10(a), and -L26(a) lines. The fold-change (RQ) of gene transcript in transgenic plants was normalized against the mean Ct value of the GAPDH gene used as an internal control. The relative expression level of gene transcript in transgenic plants is depicted with vertical bars. Error bars represent standard deviations of three replicates. Similar results were obtained in two independent qRT-PCR experiments.


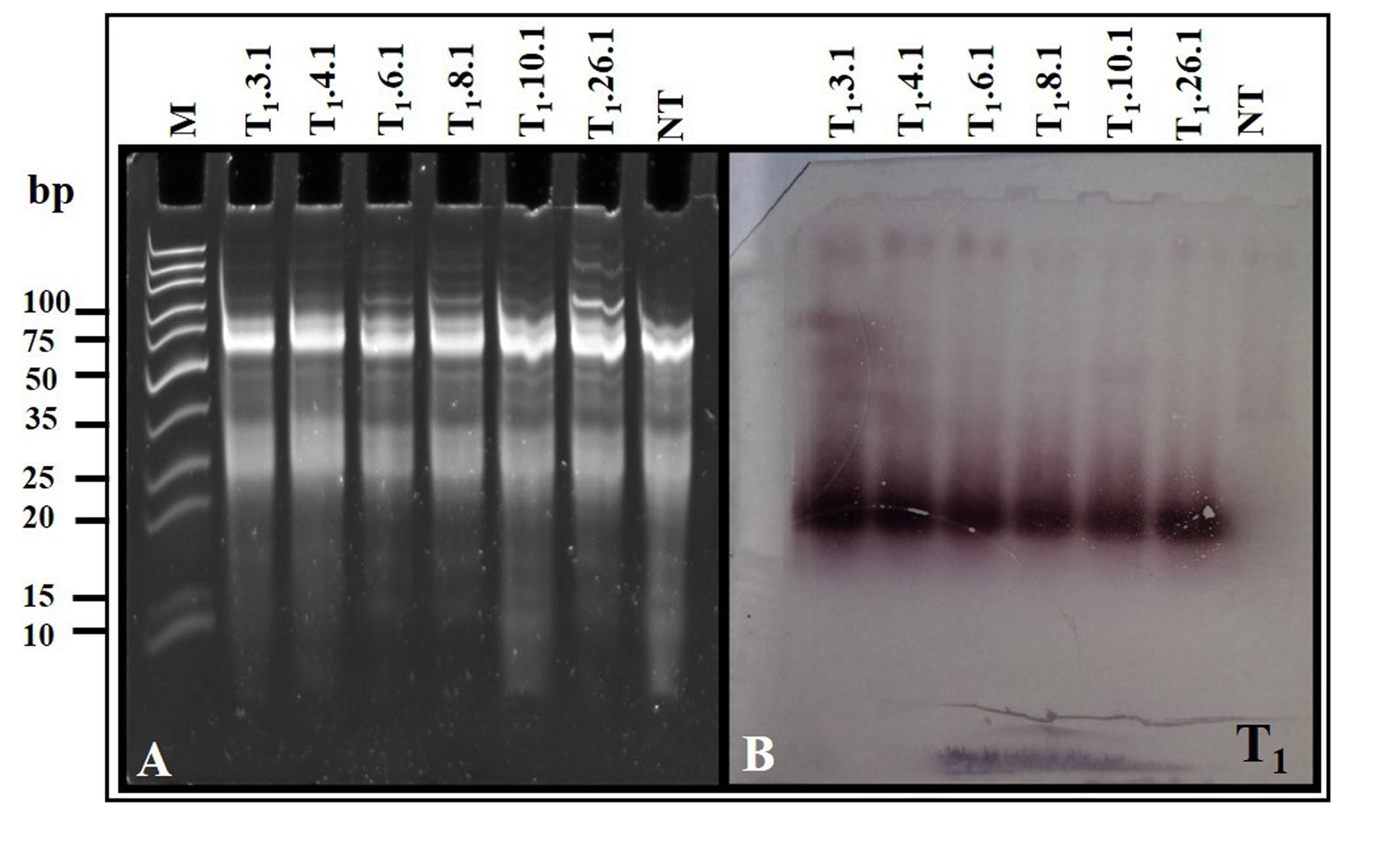


**Supplementary Figure SF4. (A)** Detection of small RNAs population of transgenic (T_1_) *G. hirsutum* cv. HS6 T_1_.3.1, T_1_.4.1, T_1_.6.1, T_1_.8.1, T_1_.10.1, T_1_.26.1 and non-transformed (NT) control plant resolved through denaturing polyacrylamide gel electrophoresis. **(B)** Northern blot analysis of siRNAs generated in transgenic T_1_ lines transferred onto HybondN membrane and hybridized with DIG-11-dUTP-labelled *C*4-derived probe.


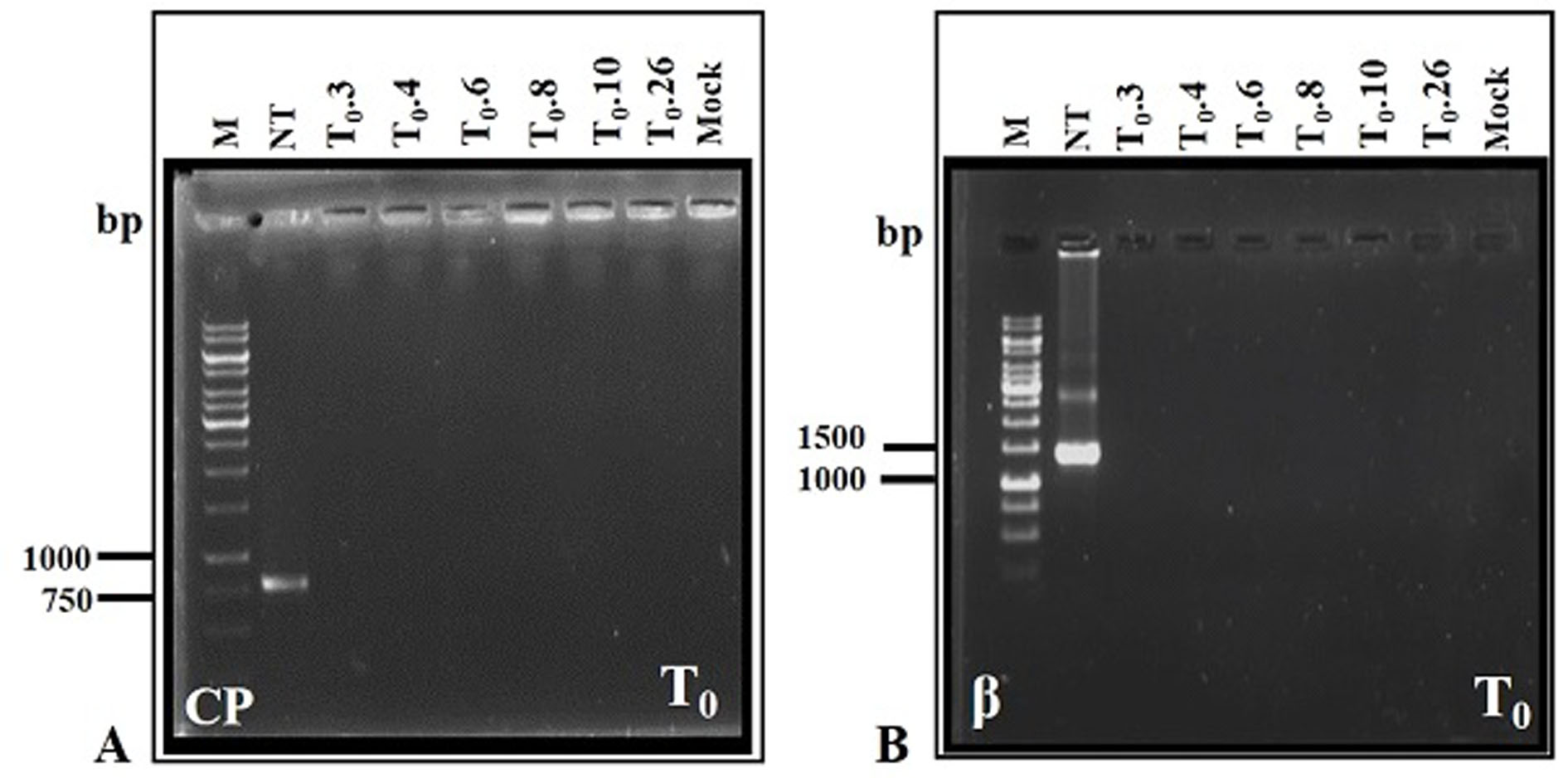


**Supplementary Figure SF5.** PCR-based detection of: **(A)** CLCuMuV-coat protein, and **(B)** CLCuMuB in transgenic (T_0_) *G. hirsutum* cv. HS6 lines T_0_.3, T_0_.4, T_0_.6, T_0_.8, T_0_.10, T_0_.26 and non-transformed control plants after eight weeks of inoculation with viruliferous whiteflies (*Bemisia tabaci*). Oligo primers specific for the CLCuMuV-CP gene and CLCuMuB were employed in PCR. Amplification of the CP gene (~780 bp) and CLCuMuB (~1.3 kb) was observed in non-transformed control plants. M is 1kb DNA marker, NT is non-transformed control, and Mock is uninoculated control.


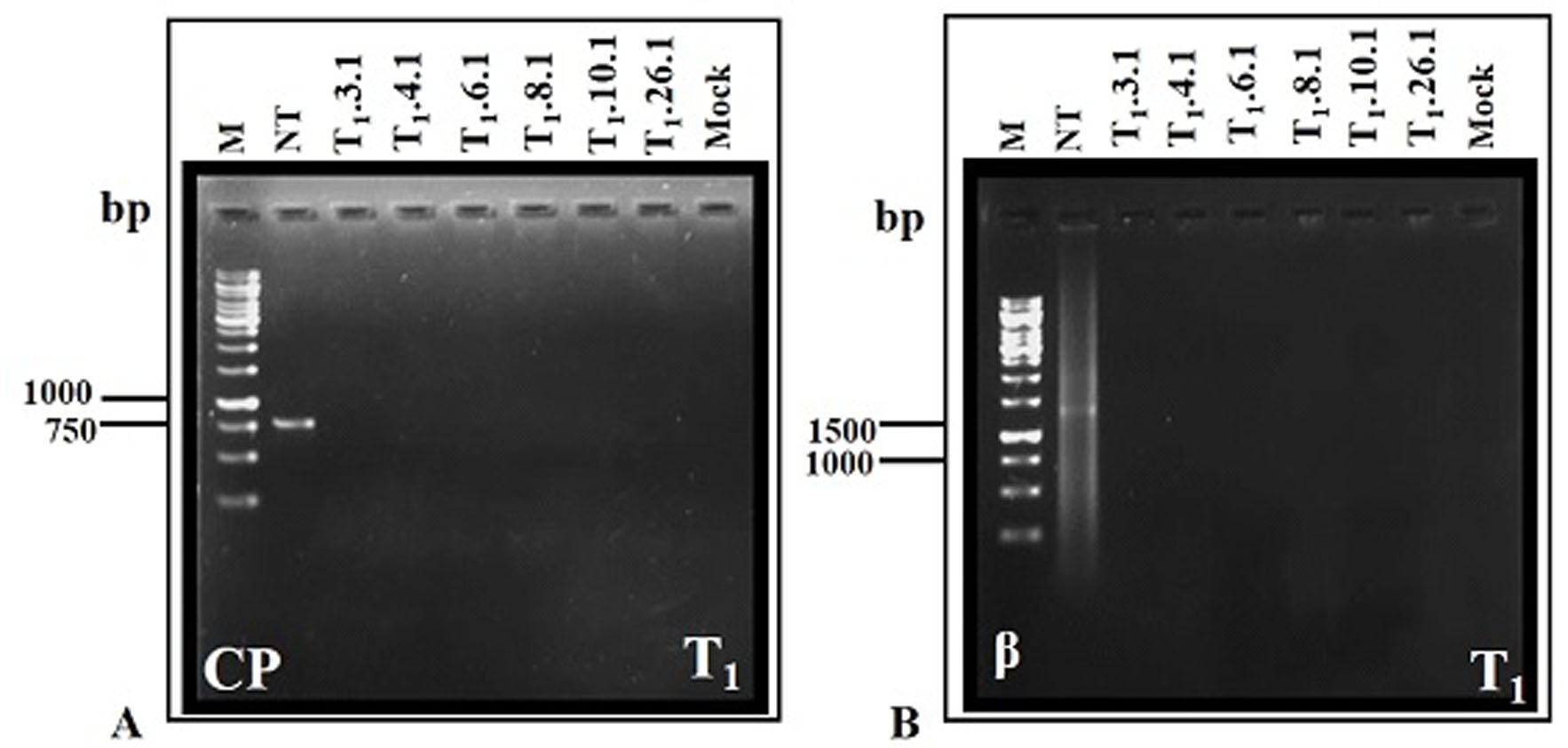


**Supplementary Figure SF6.** PCR-based detection of **(A)** CLCuMuV-coat protein (CP), and **(B)** CLCuMuB in transgenic (T_1_) *G. hirsutum* cv. HS6 lines T_1_.3.1, T_1_.4.1, T_1_.6.1, T_1_.8.1, T_1_.10.1, T_1_.26.1 and non-transformed control plants after eight weeks of inoculation with viruliferous whiteflies (*Bemisia tabaci*). Oligo primers specific for the CLCuMuV-CP gene and CLCuMuB were employed in PCR. PCR amplified CP gene (~780 bp) and betasatellite (~1.3 kb) were observed in only non-transformed control plants. M is 1kb DNA marker, NT is non-transformed control, and Mock is non-inoculated control.


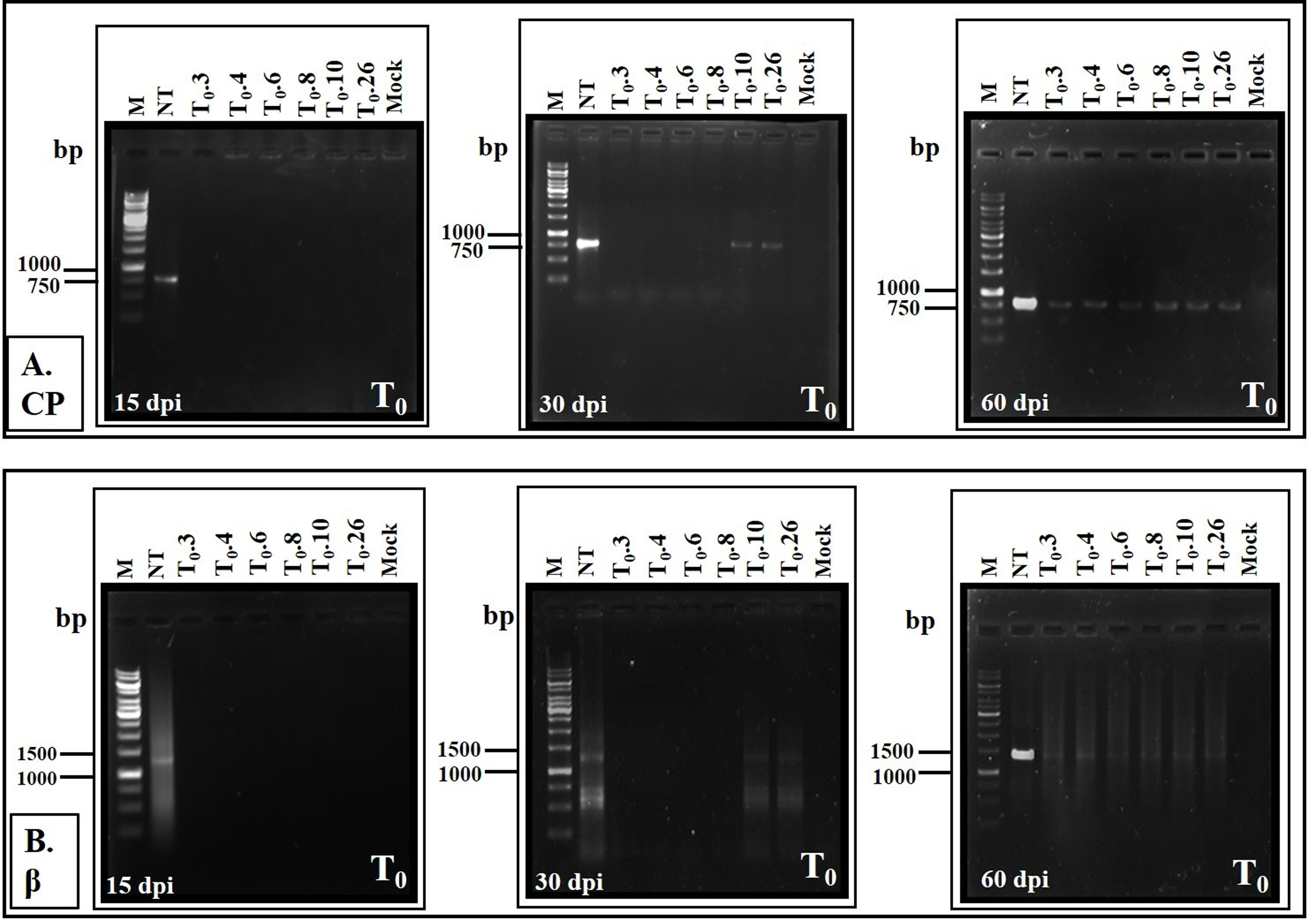


**Supplementary Figure SF7.** RCA-PCR based detection of: **(A)** CLCuMuV-coat protein (CP), and **(B)** CLCuMuB. Total genomic DNA was isolated from transgenic (T_0_) *G. hirsutum* cv. HS6 lines T_0_.3, T_0_.4, T_0_.6, T_0_.8, T_0_.10, T_0_.26 lines and non-transformed control plants at 15, 30 and 60 days after inoculation with viruliferous whiteflies (*Bemesia tabaci*) and RCA was performed. The RCA product was subjected to PCR employing primers specific for CLCuMuV-CP and betasatellite. Amplification of the CP gene (~780 bp) and betasatellite (~1.3 kb) was observed in non-transformed control plants at 15 days post-inoculation (dpi). A low degree amplification of the CP gene and corresponding betasatellite was detected in T_0_.10 and T_0_.26 lines at 30 dpi as compared to non-transformed control plants. A low degree amplification of the CP gene and betasatellite was detected in all the transgenic lines at 60 dpi as compared to non-transformed control plants. M is 1kb DNA marker; NT is non-transformed control and Mock is non-inoculated control plant.


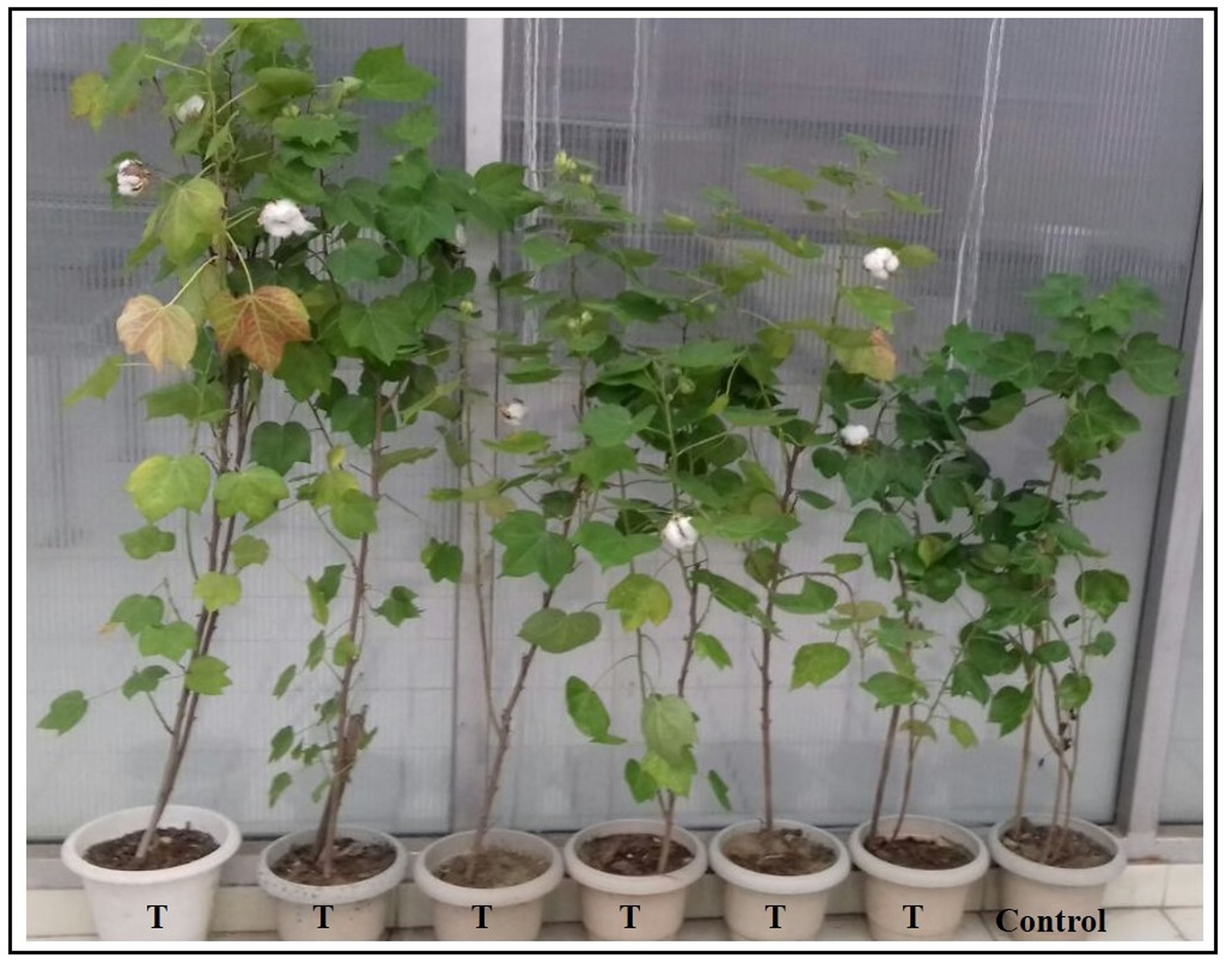


**Supplementary Figure SF8.** *G. hirsutum* cv. HS6 T_0_ transgenic (T) and non-transformed control plants at reproductive stage under greenhouse conditions showing blossom of cotton bolls. After the selfing of transgenic plants, cotton bolls were picked at maturity.

**Supplementary Table ST1:** List of oligo primer sequences used for PCR and Real Time PCR experiments

| Primer Pairs | Primer sequences |
| --- | --- |
| *C4* For  *C4* Rev | 5՛-ACTAGTGGCGCGCCGCTAGTTCCTTAATGACTCTAAGAGC-3՛ |
|  | 5՛-GGATCCATTTAAATCTGCAGAGAGCTTCACGAAG-3՛ |
| *hpt*For  *hpt*Rev | 5՛- CTATTCCTTTGCCCTCGGACG- 3՛ |
|  | 5՛- GAAAAAGCCTGAACTCACCGC- 3՛ |
| RT-*GA* For  RT-*GA* Rev | 5՛- ACCCTTCTGTTTGGCGAGAA- 3՛ |
|  | 5՛- GTCTCGCCCCATGGAATCTC- 3՛ |
| RT-*C4* For  RT-*C4*Rev | 5ʹ- GAGCCTCCGACTTACTTCC- 3՛ |
|  | 5ʹ- CAGATCGACGGAAGGTCAG- 3ʹ |
| *CP*For  *CP* Rev | 5՛- TAATATCAATTCGTTACAGAG- 3՛ |
|  | 5՛- AATTATGTCGAAGCGAGCTG- 3՛ |
| Beta For  Beta Rev | 5՛-ACCACTACGCTACGCAGCAGCC-3՛ |
|  | 5՛-TACCCTCCCTCCCAGGGGTACGC-3՛ |

For: forward primer; Rev: reverse primer. Restriction enzyme sites of *Spe*I/*Asc*I in *C4* For and *BamH*I/*Swa*I in *C4* Rev primers are underlined.

**Suppl Table ST2.** Ratio of germinated and non-germinated seeds of transgenic (T_0_) *G. hirsutum* cv. HS6 lines

| ***G. hirsutum* cv. HS6 lines** | **Number of seeds germinated (HygR)** | **Number of seeds non-germinated (HygS)** | **HygR: HygS** |
| --- | --- | --- | --- |
| HS6-L3 | 9 | 3 | 3:1 |
| HS6-L4 | 13 | 4 | 3.2:1 |
| HS6-L6 | 10 | 3 | 3.3:1 |
| HS6-L8 | 12 | 4 | 3:1 |
| HS6-L10 | 8 | 3 | 2.6:1 |
| HS6-L26 | 9 | 4 | 2.2:1 |
